# Herpes simplex virus-1 entrapped in *Candida albicans* biofilm displays decreased sensitivity to antivirals and UVA1 laser treatment

**DOI:** 10.1186/s12941-017-0246-5

**Published:** 2017-11-14

**Authors:** Cristian Ascione, Arianna Sala, Elham Mazaheri-Tehrani, Simona Paulone, Beniamino Palmieri, Elisabetta Blasi, Claudio Cermelli

**Affiliations:** 1 Department of Diagnostic, Clinic and Public Health Medicine, University of Modena and Reggio Emilia, Via del Pozzo 87, 41125 Modena, Italy; 2 Iranian Research Center for HIV/AIDS, Tehran University of Medical Sciences, Tehran, Iran; 3 Department of General Surgery and Surgical Specialties, University of Modena and Reggio Emilia Medical School, Surgical Clinic, Via del Pozzo 87, 41125 Modena, Italy

**Keywords:** Biofilm, *Candida albicans*, Human herpesvirus type-1 (HSV-1), UVA, Acyclovir, Foscarnet, Laser, Virus

## Abstract

**Background:**

Recently, we published data suggesting a mutualistic relationship between HSV-1 and *Candida. albicans*; in particular: (a) HSV-1 infected macrophages are inhibited in their anti-Candida effector function and (b) Candida biofilm protects HSV-1 from inactivation. The present in vitro study is aimed at testing the effects of Candida biofilm on HSV-1 sensitivity to pharmacological and physical stress, such as antiviral drugs (acyclovir and foscarnet) and laser UVA1 irradiation. We also investigated whether fungus growth pattern, either sessile or planktonic, influences HSV-1 sensitivity to antivirals.

**Methods:**

Mature Candida biofilms were exposed to HSV-1 and then irradiated with laser light (UVA1, 355 λ). In another set of experiments, mature Candida biofilm were co-cultured with HSV-1 infected VERO cells in the presence of different concentrations of acyclovir or foscarnet. In both protocols, controls unexposed to laser or drugs were included. The viral yield of treated and untreated samples was evaluated by end-point titration. To evaluate whether this protective effect might occur in relation with a different growth pattern, HSV-1 infected cells were co-cultured with either sessile or planktonic forms of Candida and then assessed for susceptibility to antiviral drugs.

**Results:**

UVA1 irradiation caused a 2 Log reduction of virus yield in the control cultures whereas the reduction was only 1 Log with Candida biofilm, regardless to the laser dose applied to the experimental samples (50 or 100 J/cm^2^). The presence of biofilm increased the IC_90_ from 18.4–25.6 J/cm^2^. Acyclovir caused a 2.3 Log reduction of virus yield in the control cultures whereas with Candida biofilm the reduction was only 0.5 Log; foscarnet determined a reduction of 1.4 Log in the controls and 0.2 Log in biofilm cultures. Consequently, the ICs_50_ for acyclovir and foscarnet increased by 4- and 12-folds, respectively, compared to controls. When HSV-1 was exposed to either sessile or planktonic fungal cells, the antiviral treatments caused approximately the same weak reduction of virus yield.

**Conclusions:**

These data demonstrate that: (1) HSV-1 encompassed in Candida biofilm is protected from inactivation by physical (laser) and pharmacological (acyclovir or foscarnet) treatments; (2) the drug antiviral activity is reduced at a similar extent for both sessile or planktonic Candida.

## Background

In most natural environments, microorganisms exist predominantly as biofilms rather than as planktonic cells [1]. Growing as a biofilm provides microorganisms with a plethora of advantages. During biofilm formation, microorganisms characteristically display a phenotype that is markedly different from that of their planktonic counterpart, contributing to a reduced sensitivity to antimicrobial drugs and to host’s immune response [2–8]. In clinical setting, biofilm represents an ever-growing problem accounting for up to approximately 65% of infections, particularly severe in immunocompromised hosts [2].

Recently, *Candida albicans* biofilm has gained prominence because of the increase in infections related to indwelling medical devices representing suitable surfaces for biofilm formation [9, 10]. These localized infectious foci can allow fungal cell detachment and dispersal, causing deep tissues candidiasis and candidemia, both associated with high mortality rates (30–50%) [11, 12]. The ability of *C. albicans* to form biofilm has a great impact on its pathogenicity; given the dramatically increased resistance to antifungal agents, such as fluconazole and amphotericin, biofilm-related infections are difficult to eradicate [13].

It is reasonable to envisage that in vivo, especially in anatomical sites naturally harboring an abundant and complex resident microbiota, such biofilms likely occur as poly-microbial multi-layered network of different microorganisms, experiencing both synergistic and antagonistic relationships. Recently, by an in vitro model, we demonstrated that herpes simplex virus 1 (HSV-1) and coxsackie virus B5 can be efficiently entrapped in and protected by Candida biofilm [14]; moreover, literature reports show that HSV-1 enhances *C. albicans* adherence, biofilm formation and resistance to host-mediated antifungal defenses [15, 16]. Such synergistic interactions between HSV-1 and *C. albicans* provide the rationale for the present in vitro study aimed at testing the effects of fungal biofilm on virus biology in terms of sensitivity to antiviral drugs and laser irradiation. We also investigated whether fungus growth pattern, either sessile or planktonic, influences HSV-1 sensitivity to antivirals.

## Methods

### *Candida albicans*

The *C. albicans* clinical isolate 50vr, used in the present study, was previously characterized as biofilm producer and highly virulent, as assessed by an in vivo infection model in *Galleria mellonella* [17]. *C. albicans* was kept in stock at − 20 °C and maintained for experiments by bi-weekly passages on Sabouraud Dextrose Agar plates. Fresh cultures were set the day before each experiment.

### HSV-1

The HSV-1 strain used in this work was a clinical isolate, identified by monoclonal antibodies, laboratory adapted through serial passages (> 50) on VERO cells [14, 15]. The virus inocula employed in the experiments consisted of cell-free virus suspensions, obtained from centrifuged lysates of virus-infected VERO cells. Virus batches were titrated on VERO cells (10^8^ PFU/mL) and kept frozen in aliquots at − 80 °C.

### Cell line

The VERO cell line was used for the all experiments. Cells were cultured at 37 °C and 5% CO_2_ in minimum essential medium (MEM) with 10% (growth medium) or 5% (maintenance medium) foetal bovine serum (FBS), penicillin (100 U/mL), streptomycin (100 μg/mL), ciprofloxacin (100 μg/mL) and l-glutamine (2 mM). The cell line was maintained by passages in fresh medium twice a week.

### Laser source

The Laser Alba 355 (Elettronica Valseriana, Casnigo, BG, Italy) was used as UVA1 laser source; it works at 355 λ, allowing to set different programs by combining parameters such as laser power, time of exposition, distance from the laser source and shape of the radiated area.

### Antiviral drugs

Two antiviral molecules were assessed against HSV-1, acyclovir (Recordati SpA, Milano, Italy) and foscarnet (Clinigen, Burton-on-Trent, UK). Both were commercial products commonly used for intravenous treatment.

### Biofilm formation and exposure to HSV-1

Candida cells were grown overnight at 37 °C in yeast peptone dextrose (YPD), then harvested and washed with phosphate-buffered saline (PBS). After resuspension to 1 × 10^6^ yeast cells/mL in MEM-10% FBS, 100 µL were seeded in duplicate in polystyrene, flat-bottom 96-well cell culture plates (Euroclone S.p.A., Pero-Mi, Italy) and incubated at 37 °C to allow biofilm formation, according to reported studies [14, 18, 19]. Twenty-four hours later, virus inoculum (50 μL, 10^7^ PFU/mL final concentration) was added to biofilm-containing wells and to empty control samples. The samples were incubated for additional 24 h and then exposed to either physical or pharmacological treatments. Finally, the wells were scraped for 1′ with a plastic tip and the load of infectious virus embedded in the detached/rescued biofilm was titrated (see below). Each experiment was repeated 3–4 times and each condition was tested in triplicate.

In a further set of experiments aimed at assessing whether drug antiviral efficacy may be different when Candida is cultivated as biofilm or as planktonic, Candida was also seeded in wells with a glass cover slide on the bottom. In our hands, glass inhibits the Candida strain we used (50vr) in biofilm formation: in fact, Candida 50vr when cultured on glass surfaces grow in a planktonic pattern (personal observation) with an hyphal mass floating in the culture medium.

### Virus titration

In each experiment, HSV-1 residual titer was established by end-point titration. At the end of each experiment, plate well content was harvested by scraping for 1′ with a plastic tip. After centrifugation, the rescued material was diluted with maintenance medium on a tenfold basis and each dilution was seeded in duplicate onto 24 h-old VERO cell cultures. After a 3 day incubation at 37 °C, the virus titer of each sample was established as the highest dilution showing the typical viral cytopathic effect. The results, expressed as tissue culture infectious dose 50 (TCID_50_)/mL, were calculated using the Reed and Muench formula [20].

In order to determine the Inhibitory Concentration 90 (IC_90_) or 50 (IC_50_) of the different treatments, the plaque reduction assay was used and it was performed according to published procedures [21]. After centrifugation of the rescued material, VERO cell monolayers were infected with tenfold serial dilutions of such material. After 1 h of incubation at 37 °C, virus *inoculum* was removed and each well was added with maintenance medium containing human γ-globulin anti-HSV-1 at 0.6% to neutralize non-penetrated virus. Medium was removed 2 days later, and the infected cell monolayers were fixed with methanol and stained with crystal violet (CV) to count the cytolysis plaques: in this case, virus titer was expressed as plaque forming units (PFU/mL).

### Laser treatment of *C. albicans*

The effects of UVA1 were evaluated on *C. albicans* to determine whether the laser energy could have an inhibitory activity on biofilm. In particular, to test the effects of the laser beam on biofilm formation, Candida cells were exposed to the laser beam immediately after seeding the yeast cells in culture medium (pre-treatment) and then plates were incubated for 48 h to allow biofilm formation. Also, the effects of laser irradiation were evaluated on biofilm maintenance, by exposing mature biofilm to laser beam, namely 48 h after cell seeding (post-treatment). Nine different emission protocols, with energy ranging between 20 and 250 J/cm^2^, were applied. In any protocol tested, the laser beam had a square application and the tissue culture plate was set at 30 cm from the laser beam source. The metabolic activity and the total biomass of *C. albicans* treated and untreated biofilm were quantified by XTT assay and CV staining assay, respectively.

### Crystal violet staining assay

CV staining assay was used to quantify the total biomass of control and laser-exposed *C. albicans* biofilms [22]. Briefly, wells containing Candida biofilm or controls (medium only) were washed 3 times with 200 µL of PBS and then air dried for 5′. After fixation with 100 µL of methanol for 20′, the samples were stained with 100 µL of 1% CV solution for 5′. Afterwards, each well was washed 3 times with 200 µL of distilled water and added with 33% acetic acid (100 µL/well). After 10 min, the optical densities (OD) were measured at 540 nm by a microplate reader (Sunrise, Tecan Group Ltd, Männedorf, Switzerland).

### XTT assay

The XTT colorimetric tetrazolium assay was used to evaluate the effects of laser treatments on total metabolic activity of *C. albicans* biofilm [23, 24]. A commercial kit (AppliChem GmbH, Darmstadt, Germany) was employed following the manufacturer’s instructions. Briefly, wells containing Candida biofilm or control wells (medium only), exposed or not to the laser treatments (as above described), were washed 3 times with 200 µL of PBS and then 100 µL of the colorimetric solution were added. After 2 h incubation in darkness at 37 °C, the absorbance of the colored reduction product was measured by a spectrophotometer (Sunrise, Tecan Group Ltd, Männedorf, Switzerland) at an OD of 450 nm.

### Assessment of virus sensitivity to laser treatment in the presence or absence of Candida biofilm

On the basis of XTT and CV assays results (see below), two laser protocols were employed: program A (sub-inhibiting treatment on Candida) which dispensed 50 J/cm^2^ and program B (treatment associated with a modest cytotoxicity on Candida) which dispenses 100 J/cm^2^. After 24 h incubation at 37 °C with virus, Candida biofilms were exposed or not to laser beam. Afterwards, washing and scraping for 1′ were performed and the load of infectious virus in the detached biofilm was determined by end-point titration on VERO cells. The same treatment was carried out also in culture wells without *C. albicans* (controls). The IC_90_ of UVA1 treatment on HSV-1 was determined with or without Candida biofilm. Thus, after 24 h of incubation at 37 °C and 5% CO_2_, biofilm and control cultures exposed to HSV-1 were treated with increasing laser fluencies from 10 to 60 J/cm^2^. Afterwards, the amount of residual HSV-1 in supernatants of detached materials was assessed by plaque reduction assay as detailed above.

### Assessment of virus sensitivity to antiviral drugs in the presence or absence of Candida biofilm

Twenty-four hours old biofilms were exposed to VERO cells (2 × 10^5^ cells/well) infected 24 h earlier with HSV-1 (multiplicity of infection: 30 PFU/cell); the co-cultures were performed in growth medium in the absence or presence of 50 µM acyclovir or 600 µM foscarnet. In parallel wells (with no Candida biofilm), HSV-1 infected VERO cells were treated with the same doses of the two antivirals. After 24 h of incubation at 37 °C, plates were frozen and thawed, then the viral titer of each well was determined by end-point titration on VERO cells.

In another set of experiments aimed at determining drug IC_50_, twofold dilutions of each drug were added to wells with infected VERO cells in the presence and in the absence of Candida biofilm. The viral yield in each group was titrated 24 h later by plaque assay. For acyclovir, concentrations ranging from 3 to 50 µM were assayed, while for foscarnet from 0.019 to 2.4 mM. A dose–response curve was then elaborated and the IC_50_ was determined for each drug, with or without Candida biofilm.

### Statistical analysis

The data reported in figures are the mean values (± standard deviation) from at least three different experiments performed. The results were analyzed by the two-tailed Student’s t test and were considered significant when p < 0.05.

## Results

### Laser radiation impact on Candida biofilm

**Fig. 1:**
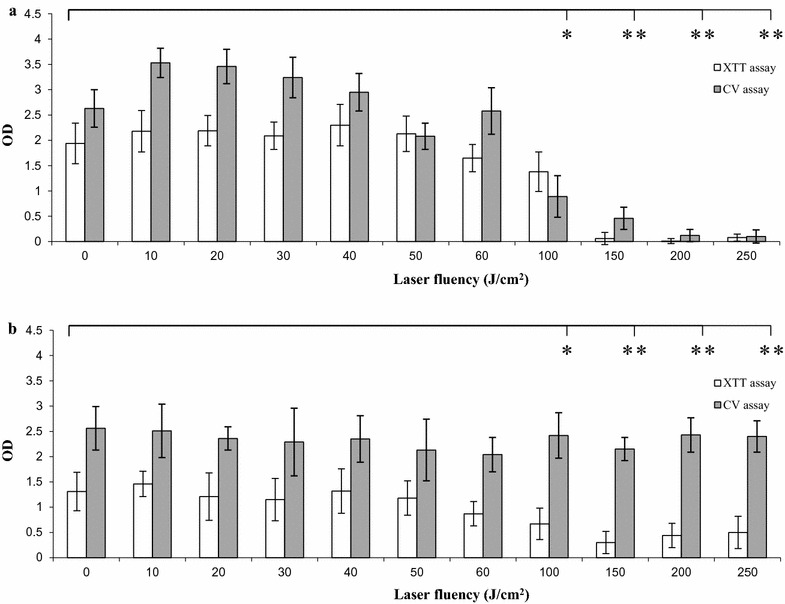
Effect of laser treatments on *C. albicans.* Effects of UVA1 light on formation (**a**) and maintenance (**b**) of *C. albicans* biofilm. Candida cells were exposed to the laser beam immediately after seeding in multiwell plates. The cultures were incubated for 48 h at 37 °C and then XTT assay and CV staining assay were carried out (**a**). In other sets of experiments, 48 h old Candida biofilms were exposed to the laser beam and then XTT assay and CV staining assay were performed (**b**). *p = 0.06; **p < 0.05

Initially, the effects of laser treatment on biofilm formation or maintenance of mature biofilm were investigated. Accordingly, Candida cells were treated with different doses of UVA1 either just after seeding onto cell culture plates, namely before biofilm formation (pre-treatment), or after 48 h of growth as biofilm (post-treatment). In both cases, the residual metabolic activity and the total biomass were assessed by XTT and CV assay, respectively. The results obtained are shown in Fig. 1 (panel a = pre-treatment, panel b = post-treatment). The OD values of biofilm exposed to laser were similar to those of unexposed cultures for treatments up to a fluency of 60 J/cm^2^ regardless of the fact that a pre- or a post-treatment had been performed. In contrast, starting from 150 J/cm^2^ fluency, laser exposure caused a reduction in OD values, with major decrease being observed when using pretreatment (upper panel) irrespective of the assay performed; differently, only metabolic activity (as assessed by XTT) but not total biomass (as assessed by CV assay) were impaired by laser treatment according to the Protocol B. Finally, 100 J/cm^2^ fluency caused an OD value reduction with a border line significance (p = 0.06) both in pre-treatment and post-treatment (in this case only for XTT values). These results indicate that a low intensity laser radiation (≤ 60 J/cm^2^) significantly affects neither biofilm formation nor its maintenance.

### Candida biofilm impact on laser antiviral activity

On the basis of the results obtained by laser radiation on Candida, two laser conditions were employed to carry out the experiments on antiviral activity of laser treatment: protocol A which dispenses 50 J/cm^2^ (sub-inhibiting against biofilm) and protocol B which dispenses 100 J/cm^2^ (the lowest fluency exerting a mild anti-biofilm activity).

**Fig. 2:**
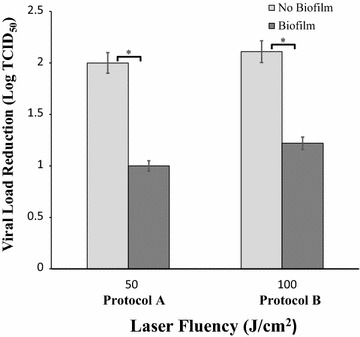
Effect of biofilm presence on virus sensitivity to laser treatment. Effects of UVA1 at fluencies of 50 and 100 J/cm^2^ on infectivity of HSV-1 free virus particles in the presence and in the absence of *C. albicans* biofilm. Twenty-four hours old Candida biofilms were exposed to HSV-1. After 24 h incubation, each sample was exposed to the laser treatment and then the culture wells were thoroughly washed, scraped and the amount of the residual virus end-point titrated. Controls (no biofilm) were run in parallel. *p < 0.05

In order to evaluate whether virus interaction with Candida biofilm can influence antiviral activity of laser beam, Candida biofilms co-cultured with HSV-1 infected cells for 24 h were exposed or not to laser beam and then the residual load of infectious virus in the biofilm was determined. The results are depicted as viral load reduction in Fig. 2. In control cultures without biofilm, protocol A reduced the amount of HSV-1 by 2 Logs (3.1 TCID_50_ vs 1.1 TCID_50_ for untreated and treated cultures, respectively), while in biofilm cultures the decrease was of 1 Log (3.1 TCID_50_ vs 2.1 TCID_50_ for untreated and treated cultures, respectively). Similar results were obtained applying protocol B: in this case, in the absence of biofilm, virus titer decreased from 3.1 TCID_50_ to 1 TCID_50_, whereas in biofilm cultures the virus titers were 3.1 TCID_50_ vs 1.9 TCID_50_ for untreated and treated cultures, respectively.

The anti-HSV-1 IC_90_ values of laser treatment in the two conditions were ascertained by treating suspensions of HSV-1 in the presence or absence of Candida biofilm with increasing laser fluencies between 10 and 60 J/cm^2^ and then titrated by plaques assay. The results showed an increased value in the presence of biofilm (18.4 J/cm^2^ vs 25.6 J/cm^2^).

### Candida biofilm impact on antiviral drug activity

**Fig. 3:**
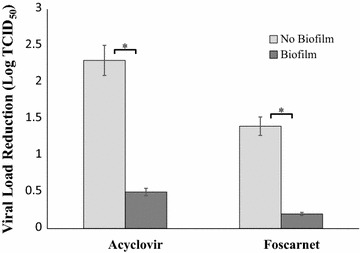
Effect of biofilm presence on virus sensitivity to antiviral drugs. Antiviral effect of 50 µM acyclovir and 600 µM foscarnet on HSV-1 infected cells embedded or not in *C. albicans* biofilm. Twenty-four hours old Candida biofilms were co-cultured with HSV-1-infected VERO cells in medium containing acyclovir or foscarnet. After 24 h incubation, samples were frozen and thawed to lyse infected VERO, then the released virus was end-point titrated. Controls (no biofilm) were run in parallel. *p < 0.05

Next, the influence of Candida biofilm on the antiviral activity of acyclovir and foscarnet was assessed. To this purpose, HSV-1 infected VERO cells were incubated with or without Candida biofilm in the presence or absence of antiviral drug for 24 h. The residual viral load was evaluated and the results, shown as viral load reduction, are shown in Fig. 3. The virus titers of VERO control cultures (no biofilm) decreased by 2.3 Log (from 3.7 TCID_50_ to 1.4 TCID_50_) when exposed to acyclovir and only by 0.5 Log (from 1.6 TCID_50_ to 1.1 TCID_50_) if the treatment had been performed in the presence of Candida biofilm. Similarly, using foscarnet, the virus titers decreased by 1.4 Log in control cultures without biofilm (from 4.5 TCID_50_ to 3.1 TCID_50_) and only by 0.2 Log when the treatment had been performed in the presence of Candida biofilm (from 3.1 TCID_50_ to 2.9 TCID_50_).

In order to establish the IC_50_ values of the 2 drugs, HSV-1 infected VERO cells were treated with increasing doses of the two antivirals in the presence or absence of Candida biofilm. Then residual HSV-1 infectivity was assessed with plaque assay. We found that acyclovir IC_50_ value increased fourfold, being 5.41 and 22.62 µM, in the absence and in the presence of Candida biofilm, respectively. Similar results were obtained for foscarnet: the IC_50_ value raised from 54 µM in the absence of biofilm to 661 µM in VERO cultures co-incubated with Candida biofilm. In this case, the increase was 12-folds.

### The reduction in drug antiviral activity observed does not depend on Candida pattern growth

Lastly, we evaluated whether the observed reduction in acyclovir and foscarnet efficacy against HSV-1can be ascribed to fungus growth manner. Thus, HSV-1 infected VERO cells were exposed to *C. albicans* grown in two different patterns: as a biofilm or planktonic, namely, in the absence or presence of a glass slide in the culture well. By direct microscope observation, we observed that when the Candida strain we used (50vr) [17] was cultured on a cover glass, only planktonic but not sessile Candida was observed.

**Fig. 4:**
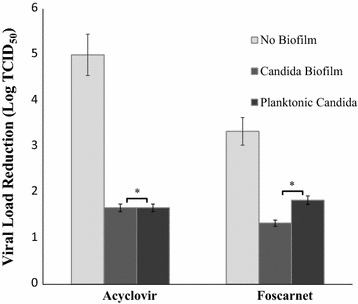
Effect of sessile and planktonic Candida on antiviral drug activity. Antiviral effect of acyclovir and foscarnet on HSV-1 infected cells embedded or not (control) in *C. albicans* biofilm and also on infected cells incubated in the presence of planktonic *C. albicans* grown on a glass slide. Twenty-four hours old Candida cells grown either with or without a glass slide on the well bottom were co-cultured with HSV-1 infected VERO cells in acyclovir or foscarnet containing medium. After 24 h incubation, samples were frozen and thawed to lyse infected cells, then the released virus was end-point titrated. Controls (no biofilm) were run in parallel. *p > 0.05

Co-cultures of Candida and HSV-1 infected cells were exposed to acyclovir and foscarnet for 24 h and the viral loads of the different cultures were then titrated. Figure 4 shows these results, depicted as viral load reduction. In control cultures without Candida, the antiviral treatments caused a reduction of 4 Logs (from 5.8 TCID_50_ to 0.8 TCID_50_) and 3.3 Logs (from 5.8 TCID_50_ to 2.5 TCID_50_) for acyclovir and foscarnet, respectively. In contrast, in cultures with Candida, the virus titers decreased by about 1 Log with either drug, regardless of the fact that the cover glass slide was or was not present: for acyclovir, the virus yield values were 3.3 TCID_50_ with planktonic Candida and 3.5 TCID_50_ with Candida biofilm; for foscarnet, the values were 3.8 TCID_50_ and 3.2 TCID_50_.

## Discussion

Biofilm, a major problem in clinical practice, is a structured community usually formed by different microbial types, mainly bacteria and fungi. Few studies are available on the existence of virus entrapping microbial biofilm and they focus mostly on aquatic biofilms [25]. Recently, we demonstrated in vitro that viruses, such as HSV-1 and Coxsackie Virus B5, while embedded in *C. albicans* biofilm, still retain their infectivity [14]. We also observed that such intra-biofilm localization partially reduces virus particles sensitivity to hypochlorite. By that same in vitro model, here we demonstrated that Candida biofilm protected HSV-1 also from pharmacological treatments (acyclovir and foscarnet, the most used drugs against HSV-1 infections). Moreover, we documented a virucide activity exerted by UVA1 laser and that such activity was decreased in presence of Candida biofilm.

Different hypotheses can be considered to explain such phenomena.

Firstly, a reduced antiviral drug availability might occur within biofilm, due to either drug molecules engagement by the EPS organic material or aspecific binding to fungal cells surface. Indeed, it is well known that the presence of many organic (proteins) or inorganic (electrolytes, divalent cations) substances leads to decrease or complete inhibition of disinfectant activity, both in vitro and in vivo [26, 27]: for instance, bovine serum albumin is indeed used to this purpose in studies on antimicrobial molecules. Alternatively, extracellular enzymes secreted by fungal cells in the EPS might directly interact with drug molecules, thus impairing their efficacy. Acyclovir/foscarnet activity was also tested in the presence of the same Candida strain grown as either planktonic form or biofilm. In both cases, a similar reduction in antiviral activity, with no significant difference, was observed, suggesting that EPS matrix production is not essential to affect drug activity. On these bases, drug-aspecific binding to fungal cells, followed or not by cell entry, could be the most likely hypothesis: the huge amount of biomass presents in both conditions, in one case forming a floating mycelium network and in the other a sessile biofilm structure, might represent a major hindrance to virus-drug interaction. An indirect support to this hypothesis comes from immunofluorescence studies: we observed that HSV-1 antigens or infected cells are more concentrated in areas with higher hyphae density (unpublished data). On the other hand, secretion of enzymes interacting with drug molecules cannot be ruled out: in both growth patterns, Candida excretes a plethora of molecules which could interfere with drug action.

As far as UVA1 irradiation, laser emission is currently used in medicine for topic treatment of different skin and dental diseases as well as for disinfection of surfaces, surgical instruments and water. We observed that when HSV-1 suspensions are exposed to UVA1 light at 355 λ, they undergo a significant inactivation. However, this antiviral activity is significantly affected by the presence of Candida biofilm. In particular, while HSV-1 infectivity was reduced by 2 Logs in the absence of biofilm, both at 50 or 100 J/cm^2^, infectivity was reduced by only 1 Log when biofilm was present. Consequently, IC_90_ values are 25.6 and 18.4 J/cm^2^ with and without biofilm, respectively. This reduction in drug efficacy could be likely due to energy absorption by the organic mass: although most of the biological molecules have an absorbance peak at low frequencies (210–270 λ), a lower absorbance is still present at higher frequencies (such as 355 λ) and might be sufficient to subtract energy for virus inactivation.

Evidence exists on the mechanisms of laser action on mammalian cells; in particular, UVA1 radiation induces apoptosis by two mechanisms, the one triggered by singlet oxygen species production, the other involving damages to cellular mitochondria [28]. Reactive oxygen species cause damages to fungal cell wall, lipid membranes, nucleic acids, and react with organic molecules in biofilm EPS leading to a partial/complete inactivation of such molecules: bovine albumin represents an example. As said before, it can neutralize the antimicrobial activity of chemical and physical treatments. It is a globular structured protein of high molecular mass, with a single sulfhydryl (SH) group at amino acid residues (Cys-34), which might react with free radicals [29]. Consequently, the large mass of fungal cells with its huge amount of organic target for radiated UVA light as well as the abundant organic material in the EPS might quench laser energy and subtract power, in turn reducing its efficacy on virus particles. Also heat generation by laser irradiation must be considered. As a matter of fact, a photothermolysis effect is among the mechanisms by which UVA irradiation exerts its biological activities [30]: this heating can be responsible, at least partially, for HSV-1 inactivation, considering virus fragility due to the envelope presence. The thick biofilm lime could reduce this heating effect. Finally, the UVA1 laser antimicrobial activity can be ascribed to the production of toxic molecules caused by its interaction with polystyrene of the tissue culture plate. It is well known that exposure to UVA radiation can cause significant degradation of many materials, inducing photooxidative degradation which results in breaking of the polymer chains and free radical production [31]. Again, dispersion in and sequestration by the biofilm mass could reduce the availability for virus of these antimicrobial molecules.

On the whole, our present and previous data [14] show that HSV-1 encompassed in Candida biofilm can retain its infectivity and, more important, is protected from inactivation by chemical (hypochlorite) [14], physical (laser) and pharmacological treatments. Based on these findings, we may envisage that, in vivo, *C. albicans* biofilm may represent not only a persistent reservoir of fungal cells but also of infectious virus; in fact, circulating virus particles/virus-infected cells, during viremic infections, might be retained and protected within biofilm and, later on, might be released for further dissemination. Accordingly, the presence of Candida biofilm should represent an additional health risk factor, given its ability to entrap/release also viral particles, efficiently protected against conventional antiviral treatments; such a possibility might be dramatic especially in immunocompromised patients. Finally, also the chemo-physical procedures aimed at warranting virus decontamination of materials and surfaces should be carefully reconsidered, since their efficacy might be drastically impaired by the concomitant presence of fungal biofilm.
